# Clinical features and advances in the genetics of periodic paralysis

**DOI:** 10.7717/peerj.20840

**Published:** 2026-03-03

**Authors:** Man Luo, Beibei Liu, Junjie Xu, Danyang Meng

**Affiliations:** 1 Department of Neurology, Affiliated Hospital of Jiaxing University, Jiaxing, Zhejiang, China; 2 Department of Central Laboratory, Affiliated Hospital of Jiaxing University, Jiaxing, Zhejiang, China

**Keywords:** Periodic paralysis, Paroxysmal muscle weakness, Ion channelopathy, Clinical feature, Mutant gene

## Abstract

Periodic paralysis (PP) is a group of ion channel diseases with incomplete autosomal dominant inheritance, except in sporadic patients. Ion channel gene mutations cause transient abnormalities in skeletal muscle excitability and muscle weakness. Different mutation sites cause different pathogenesis, which is very important for the classification, clinical manifestations, treatment and prognosis of periodic paralysis. Currently, the recognized mutated genes are *CACNA1S* (chromosome 1q31-32), *SCN4A* (chromosome 17q23-25), *KCNJ2* (chromosome 17q23), and *KCNJ18* (chromosome 17p11.2). The common mutation sites include R528H and R1239H in *CACNA1S*, and R672H and T704M in *SCN4A*. However, there is accumulating evidence that other mutation sites in *CACNA1S* and *SCN4A*, and even new ion channel mutations may induce periodic paralysis. Their different pathogenesis, clinical features and therapeutic measures have been widely described. This review will introduce the clinical manifestations of periodic paralysis, the different mutation sites of each ion channel, and the pathogenesis. Based on the clinical types of periodic paralysis, the characteristics of the latter are further discussed.

## Introduction

Periodic paralysis (PP) is a rare inherited neuromuscular disorder, except in sporadic patients. It is a skeletal muscle channelopathy characterized by the clinical manifestations of paroxysmal skeletal muscle weakness and abnormal serum potassium ion levels, with normal interictal muscle strength (Farooque et al., 2020; Fialho, Griggs & Matthews, 2018). The pathogenesis of this disorder is linked to abnormalities in the functioning of three ion channels (Ca^2^^+^, Na^+^ and K^+^) in muscle membrane, which may be associated with the ion channel mutations or external factors or external factors (*e.g.*, drugs, toxic substances) (Brugnoni et al., 2022; Shankle & Keane, 1988), or secondary to the internal environment and electrolyte disturbances caused by renal and metabolic diseases (Correia et al., 2023; Jackson et al., 2021), or a combination of both (*e.g.*, hyperthyroid periodic paralysis (TPP) caused by channel mutations and hormonal disturbances) (Allard et al., 2023; Cui & Zhang, 2022; Patel & Ladak, 2021).

PP is recognizable as a primary or secondary disease based on whether it is secondary to underlying disorders. It can also be categorized as HyperPP, HypoPP, or Andersen-Tawil syndrome (ATS) based on the level of serum potassium at the time of attack, HyperPP and HypoPP can further be subclassed as familial HypoPP/HyperPP or sporadic HypoPP/HyperPP (Fialho, Griggs & Matthews, 2018; Vivekanandam, Jayaseelan & Hanna, 2023). Secondary PP is observed primarily in thyrotoxic periodic paralysis and renal tubular acidosis-triggered periodic paralysis. The clinical manifestations, diagnosis, and prognosis of PP, and the therapeutic measures for its treatment vary from one etiology to another. Therefore, the clinical and genetic specificity of the different forms of PP must continuously be explored and summarized for accurate molecular diagnosis and development of tailored treatment plans; this should provide the basis for lifestyle recommendations and genetic counseling.

This review is intended for researchers in neurology, prenatal screening, clinical practice, and genetics. Exploring the relationship between the clinical manifestations, pathogenesis and gene mutations of periodic paralysis will help to better clarify the diagnosis and treatment. Finally, with the continuous elucidation of new mutation sites and mechanisms of periodic paralysis, it is beneficial to its diagnosis and treatment and to reduce the attack of weakness. Patients can get earlier and better treatment and prevent the attack of weakness.

## Survey Methodology

**Figure 1:**
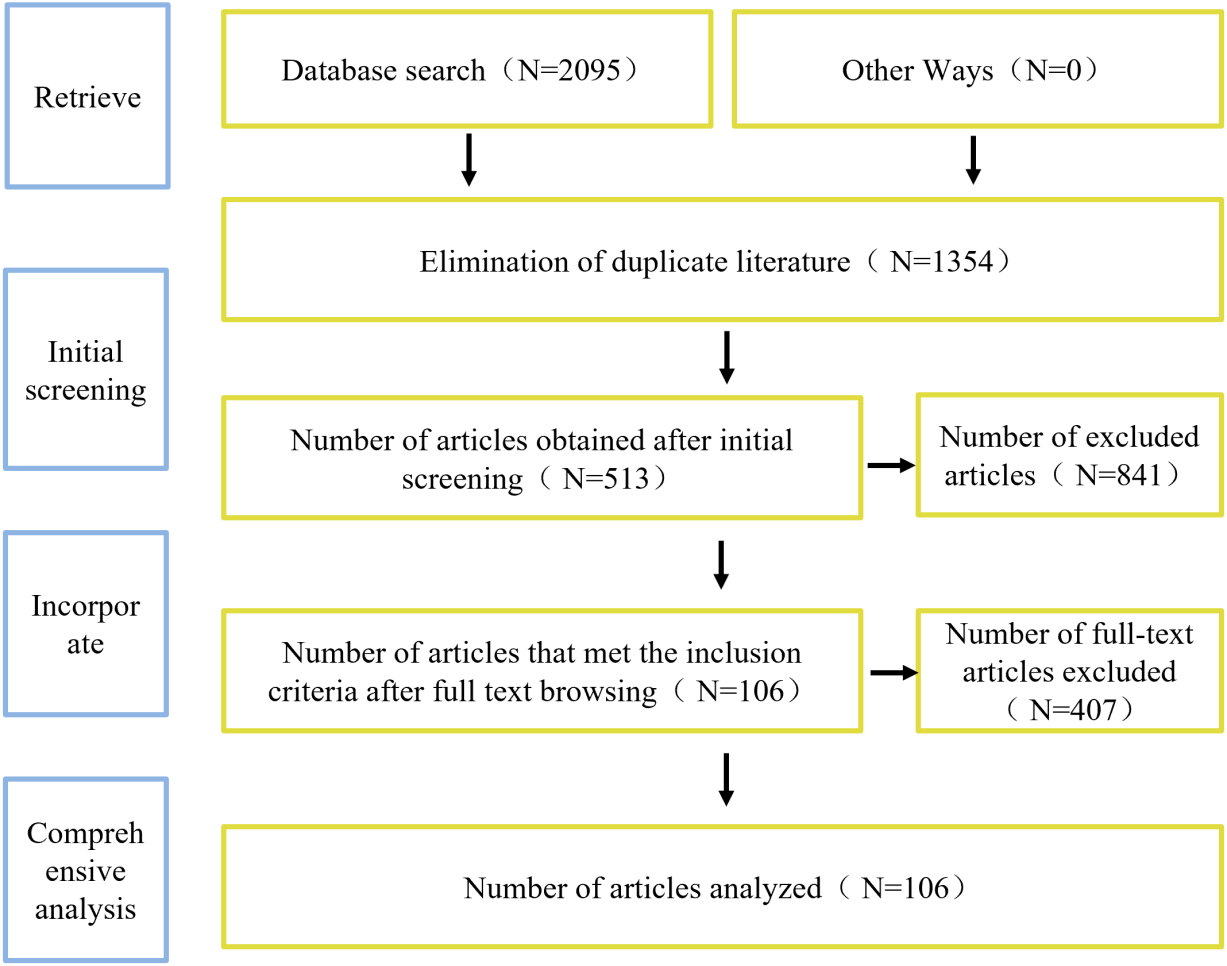
The workflow diagram of this article.

This study conducted comprehensive literature searches in the PubMed and Web of Science and Cochrane Library databases, focusing on studies published before 2024. Searches were performed using the terms “periodic paralysis (Title)”, “HyperPP or Hyperkalemic periodic paralysis (Title)”, “HypoPP or Hypokalemic periodic paralysis (Title)”, “thyrotoxic periodic paralysis or TPP (Title)”, and “Andersen-Tawil syndrome (Title)”. Duplicate articles and those not indexed in SCI were excluded, as well as those without full text availability. The key search terms “periodic paralysis and Genetic”, “HyperPP and Genetic”, “HypoPP and Genetic”, “TPP and Genetic”, “ATS and Genetic” guided the search process. The articles were screened according to the title and abstract to exclude articles not related to periodic paralysis. Further, the full text evaluation was used to exclude articles that did not meet the criteria such as the inability to obtain the full text, the presence of complications, and rare causes. The screening process was conducted independently by two investigators, and disagreements were resolved through third-party negotiation. A total of 106 literatures were included, which were composed of systematic reviews and original studies. The workflow diagram of this article is presented in Fig. 1.

### Clinical features of periodic paralysis

PP is an ion channelopathy with recurrent episodes of flaccid weakness and abnormal serum potassium levels. Common triggers of PP include post-exercise rest, stress, and alcohol consumption. Its attacks are commonly generalized weakness with proximal muscle predominance, often involving both limbs, and even affecting respiratory function, severe respiratory distress is rare during attacks, but potassium imbalance can sometimes cause cardiac arrhythmias (Farooque et al., 2020; Fialho, Griggs & Matthews, 2018; Kil & Kim, 2010).

The symptoms of familial HypoPP mostly begin during the teen years or twenties, often occurring at night or upon awakening in the early morning. Weakness caused by the disorder could be focal or generalized, but facial and respiratory muscles are rarely involved in the process, which lasts for several hours or days before gradually subsiding. The frequency of attacks varies significantly, from several times a day to only once in a lifetime. The attacks can be triggered by stress, strenuous exercise, or carbohydrate-rich foods, or they may develop spontaneously after a long rest and are preceded typically by prodromal symptoms, such as hallucinations, fatigue, and behavioral and cognitive changes. Episodes of weakness are commonly associated with low serum potassium concentrations, with fixed permanent proximal weakness independent of the frequency and severity of the attacks and not often myotonia-related (Aquino et al., 2016; Banzal et al., 2004; Finsterer, 2008; Ikeda & Okamoto, 2001; Statland et al., 2018; Weber & Lehmann-Horn, 1993).

Familial HyperPP’s first attack occurs earlier than HypoPP’s, often when the patient is still a child, it persists for 1–4 h and is a short attack. The weakness is mild and generally accompanied by myotonia. However, the frequency of these attacks increases with age, reaching peak levels in early adulthood and decreasing significantly after middle age. They commonly begin as chronic progressive myopathy that can eventually become permanent myotonia and myopathic weakness. Glucose and potassium play contrasting roles in HyperPP and HypoPP. In HyperPP, elevated potassium levels trigger episodes of weakness, and glucose improves the symptoms of skeletal muscle weakness, whereas, in HypoPP, carbohydrate-rich foods are the trigger and potassium supplementation the treatment (Farooque et al., 2020; Fialho, Griggs & Matthews, 2018; Finsterer, 2008).

ATS is a multisystem channelopathy often marked by PP, ventricular arrhythmias, and developmental abnormalities. PP features in ATS are similar to those in HypoPP in terms of the timing of the episodes, typical triggers, and muscle weakness; however, potassium ion levels can be normal or elevated during episodes (Pérez-Riera et al., 2021). Other reported manifestations of ATS include distinctive facial features, dental and skeletal abnormalities, and unique neurocognitive phenotypes with defects in executive and abstract reasoning (Nguyen, Pieper & Wilders, 2013; Totomoch-Serra, Marquez & Cervantes-Barragan, 2017).

TPP, as a neuromuscular complication of hyperthyroidism, is regularly observed in young Asian men, who account for about 5% of hyperthyroidism cases. The clinical features and triggers of TPP are similar to those of familial HypoPP, with episodes of weakness occurring only in hyperthyroidism but not during normal thyroid functioning (Allard et al., 2023). Serum potassium levels are often significantly decreased; however, the symptoms of thyrotoxicity are more pronounced in some patients and very mild in others. Even PP phenotypes can routinely indicate thyrotoxicity (Allard et al., 2023; Cui & Zhang, 2022; Patel & Ladak, 2021). Therefore, all patients with new-onset HypoPP must be examined thoroughly for thyroid functioning (Mcfadzean & Yeung, 1967). In case of abnormalities, treatment to restore the normal thyroid state must be conducted, with potassium supplemented cautiously and slowly: potassium supplementation-related improvements are not noticeable until thyrotoxicosis is under control (Abouzahir et al., 2009; Berwaerts et al., 1996; Erem, 2005; Pili et al., 2002; Zayac et al., 2016).

### Genetic, pathogenesis, and electrophysiological characteristics of periodic paralysis

As an incompletely episodic autosomal dominant disorder, four genes have so far been identified as mutations of ion channels that cause PP, CACNA1S, SCN4A, KCNJ2, and KCNJ18 which encode Ca^2+^, Na^+^, and K^+^ ion channel proteins (Cannon, 2015; Corrochano et al., 2014; Lulsegged, Wlodek & Rossi, 2011; Marrus et al., 2011; Ryan et al., 2010; Struyk & Cannon, 2007; Wu, Mi & Cannon, 2013). PP gene mutations result in the abnormal depolarization of skeletal muscles, causing the inactivation of most voltage-gated sodium channels (wild type and mutants) and non-excitable muscle fibers, which brings about episodes of myotonia and myopathic weakness (Farooque et al., 2020; Fialho, Griggs & Matthews, 2018; Venance et al., 2006). And the mutation sites have been reported in periodic paralysis was listed in Table 1.

**Table 1:** Mutation sites have been reported in periodic paralysis.

**Categorize**	**Mutation gene**	**Mutation exons**	**Mutation fragments**	**Mutation sites**
HypoPP	*SCN4A*	5, 12, 18, and 19	The S4 of II and IV	*R222W, R669H, F671S, R672H, R672G, R672C, R672S, R675Q, R1132Q, R1135H, and P1158S.*
	*CACNA1S*	11, 20, 21, 22, 26, and 30		*R528H/G,V876E, R897S, R900G/S, H916Q, R1086C, and R1239H/G*
HyperPP	*SCN4A*	13, 19, 22, and 24	S5-S6 and S4, S1 of II and IV	*T704M, R675G/E/W/Q, I693T, L689I, V781I, S906T, A1156T, Tl313M, M1360V, M1370V, L1433R, R1448C, F1490L, M1493I, I1495F, and M1592V*
ATS	*KCNJ2*			*R218W*, *R218Q*, *R67W*, *T192A*
TPP	*KCNJ18*			*K366R*, *R205H*, *Q407X*, *T354M*, *R339X*, *K360T*, *Q126X*, *E388K*, *A200P*

Periodic paralysis mutant genes determine the type of ion channel and the mode of dysfunction, thereby determining the electrophysiological and clinical characteristics of changes in myofiber membrane excitability. The electrophysiological features of periodic paralysis directly point to the mutation channel, which can significantly narrow the scope of genetic screening (Horga et al., 2013; Jin et al., 2017). Clinically, we can combine clinical manifestations and electrophysiological characteristics to achieve an efficient diagnostic pathway of targeted sequencing (Fournier et al., 2004; Ribeiro et al., 2022). TPP is a sporadic disease caused by a combination of polygenic susceptibility and thyroid hormone environment, which is different from the monogenic dominant pattern of familial PP. Particular variants of periodic paralysis have been less studied. Therefore, this article summarizes the electrophysiological characteristics and important clinical features of the common mutated genes of HypoPP, HyperPP and ATS. The summary of these relationships has been presented in the relevant studies on periodic paralysis. The specific information can be found in Table 2.

**Table 2:** Electrophysiological, clinical, and genetic relationships in periodic paralysis.

Gene (mutation hotspots)	Channels/mechanism	Electrophysiological characteristics	Clinical Features	induce
*CACNA1S* R528H/R1239H	Cav1.1*α* gated the pore current Igp	LET type I to III, CMAP↓ ≥40, Slow recovery, EMG showed no myotonia	HypoPP-1 Early onset (<10 years old), duration >24 h,vacuolar myopathy	High carbs, wee hours, stress
*SCN4A* R672H/R1132Q	NaV1.4 gated hole and inactivation left shifted	LET positive,Low temperature SET can be positive, No myotonia	HypoPP-2The onset was later, lasted less than 12 h, and had tubular aggregation	It can be aggravated by acetazolamide
*SCN4A* T704M/M1592V	NaV1.4 inactivation	SET positive, cold-induced myotonic potential, LET positive	HyperPPEpisodes <2 h, induced/aggravated by oral potassium, and myotonia	Cold add potassium that attack
*KCNJ2* R67W/R218W	Kir2.1 loss of function	EMG showed repetitive discharges, ECG showed prolonged QT and polymorphic ventricular tachycardia	ATS, Low/high/normal potassium can be, skeletal deformity	Mood/menstruation can be induced
*SCN4A* R1448H	NaV1.4 cold sensitivity	SET cold environment positive: myotonia → weakness, LET can overlap positive	PMC overlap HypoPP-2, Myotonia followed by hypokalemic muscle weakness may occur successively	A small dose of mexiletine is effective

### Hypokalemic periodic paralysis

The non-excitability of hypokalemic periodic paralyzed muscle fibers stems from abnormal depolarizing leakage currents caused by mutations in voltage-gated calcium channels or in the voltage-sensing region of sodium channels of the myofibrillar membrane. Most HypoPP cases are instigated by mutations in *CACNA1* S (HypoPP-1) and *SCN4A* (HypoPP-2) (Finsterer, 2008; Fontaine et al., 1994; Riggs, 1988; Stapleton, 2018). Approximately 70% to 80% stem from mutations in calcium channels and around 10% from alterations in sodium channels (Ryan et al., 2010). Both genes have four homologous repeating structural domains (DI-IV) in the alpha subunit and six transmembrane helical segments (S1–S6) in each domain, with the S4 segment containing positively charged arginine to form the voltage sensor and the S5–S6 segments of each structural domain forming the ion channel. The principal voltage-gated calcium channels in skeletal muscles are the slowly inactivating L-Ca^2+^ channels. There is dihydropyridine L-type calcium channel (DPH) binding site in the S5–S6 region of the III–IV structural domain of Cav1.1; these also serve as the regulatory point for second messengers and toxic effects. The intracellular loop between the II–III domains is coupled to the Ryanodine receptor, which is the excitation-contraction coupling site. Nav1.4 is an essential structure for the formation and propagation of action potentials during cell excitation, with the *α* subunit forming five parts of the functional edifice of the ion channel (ion channel pore, ion-selective region, voltage sensor, channel gate, and fast inactivation gate). Pore region, S4, and the fast inactivation gate located in the intracellular loop between III and IV affect standard selectivity and the activation and inactivation of the sodium channel. As the voltage receptor site, S4 is critical to maintaining regular channel activity.

The *CACNA1S* gene mutations of HypoPP (HypoPP-1)—*R528H/G*, *V876E*, *R897S*, *R900G/S, H916Q, R1086C, and R1239H/G*—are located in exons 11, 20, 21, 22, 26, and 30, *R528H/G* and *R1239H/G* are the most common mutations, accounting for about 70%–80% of the cases (Brugnoni et al., 2022; Wang et al., 2015; Yuan et al., 2023). HypoPP-related *SCN4A* mutations (HypoPP-2)—*R222W*, *R669H*, *F671S*, *R672H*, *R672G*, *R672C*, *R672S*, *R675Q*, *R1132Q*, *R1135H*, and *P1158S*—are located in exons 5, 12, 18, and 19, with hotspots in exon 12; *R672H* is the most common type (Kuzmenkin et al., 2002; Matthews et al., 2009; Matthews et al., 2011; Park & Kim, 2010; Sugiura et al., 2003; Wu et al., 2011; Zhang & Xiao, 2022). Concerning the location of the mutation sites, all are located in the S4 fragment, except *V876E*, which is located in the S3 fragment (Ke et al., 2009), with the S4 of II and IV being the most common cases (95%) (Burge & Hanna, 2012; Matthews et al., 2009). Regarding what is substituted only, all substitutions are arginine, except for *F671S, H916Q, P1158S,* and *V876E* substitutions. Substitution by histidine accounts for more than 95% of the cases, whereas substitution by others, like glycine, serine, and cysteine, makes up less than 5% of the cases (Matthews et al., 2009).

Interestingly, most *SCN4A* and *CACNA1S* hypoPP mutations share a similar molecular defect, and affect positively charged amino acids in the voltage sensor region (Matthews et al., 2009). Mutations in the voltage sensor region reportedly set in motion an alternative conduction pathway by monovalent cations, called the *ω* current, to distinguish it from the *α*-current conducted through the primary ion pore (Allard & Fuster, 2018; Francis et al., 2011; Nagasaka et al., 2020; Starace & Bezanilla, 2004; Starace, Stefani & Bezanilla, 1997; Tombola, Pathak & Isacoff, 2005). The same leakage current has been identified on the arginine mutations in Nav1.4 II of HypoPP. It occurs at hyperpolarized membrane potentials but causes only minimal displacement of the muscle membrane potential at rest. However, it significantly impacts the muscle membrane during hypokalemia by increasing potassium concentration and causing a paradoxical depolarization. Even slightly lower potassium levels—but still within the physiological range—can trigger abnormal depolarizations and episodes of weakness.

The common mechanism of HypoPP is that the “mutation of arginine to histidine/glycine” in the S4 segment produces gated pore current (Igp), mild depolarization at rest, and aggravation of depolarization when low potassium causes NaV inactivation, eventually leading to paralysis. The electrophysiological characteristics of HypoPP-1 caused by *CACNA1S* mutation are the most common long Exercise Test (LET)-I (CMAP decreased more than or equal to 40% for 40 min without recovery), Short Exercise Test (SET) negative and EMG without myotonia (Jin et al., 2017; Ribeiro et al., 2022; Simmons et al., 2019; Tengan et al., 2004). The clinical characteristics were early onset (<10 years old), duration of not less than 24 h, and prone to fixed proximal weakness. However, in 5–10% of *CACNA1S*-HypoPP-1 families, LET shows rapid recovery with a decrease of <40%, which is easy to be misdiagnosed as negative and needs to be confirmed by genetic screening. The electrophysiological characteristics of HypoPP-2 caused by *SCN4A* gating pore mutation were LET-II (the decrease was not less than 40%, and gradually recovered in 20–40 min), hypothermic SET occasionally positive, and EMG without myotonia (Jin et al., 2017; Simmons et al., 2019; Tan et al., 2011; Tengan et al., 2004). Clinically, the onset age is later, the duration of onset is less than 12 h, and acetazolamide can aggravate weakness.

### Hyperkalemic periodic paralysis

HyperPP-associated mutations in the SCN4A gene are located in exons 13, 19, 22, and 24, with amino acid substitutions in *R675G/E/W/Q*, *I693T*, *L689I*, *T704M*, *V781I*, *S906T*, *A1156T*, *Tl313M*, *M1360V*, *M1370V*, *L1433R*, *R1448C*, *F1490L*, *M1493I*, *I1495F*, and *M1592V*, with *T704M* being the most common type (Ammar et al., 2015; Charles et al., 2013; Corrochano et al., 2014; Fan et al., 2017; Vicino, Brugnoni & Maggi, 2023; Yuan et al., 2023). Mutations in HyperPP occur predominantly in S5–S6 and S4 segments of Nav1.4. Mutations in the S1 fragment of II and IV have also been reported.

Mutations in the *SCN4A* gene of HyperPP affect its gating function, resulting in functional defects or enhanced activation (Cummins et al., 1993; Statland et al., 2018) characterized by impaired inactivation. Continuous abnormal Na^+^ currents instigate long-duration depolarization, prompting episodes of weakness (Brown Jr, 1991). Initially, inward currents cause mild depolarization and bring about myotonia and, eventually, a deep depolarization of the myofilaments that initiates the inactivation of mutant and wild-type channels, preventing myofibers from excitation and ultimately resulting in episodes of weakness (Ammar et al., 2015; Khogali et al., 2015; Lucas et al., 2014). The disruption of slow inactivation in HyperPP mutations is associated with symptoms of PPs (Cannon, 2015), with changes that only affect gating dynamics usually linked to myotonia, *SCN4A* is also the causative gene for congenital myotonia (Vicino, Brugnoni & Maggi, 2023), but without the episodes of weakness (Cannon, 2018).

The mechanism of HyperPP is that *SCN4A* inactivation and impaired gating lead to continuous Na^+^ influx. Mild Na^+^ influx leads to depolarization and myotonia, and severe Na^+^ influx leads to depolarization and global inactivation, leading to paralysis. The electrophysiological features were positive SET/cold test (cold-induced myotonic potential followed by CMAP decrease) and positive LET with rapid recovery (Charles et al., 2013; Ribeiro et al., 2022; Simmons et al., 2019; Tan et al., 2011). The clinical features are that the seizure duration is less than 2 h, can be induced or aggravated by oral potassium, and cold stimulation is most easily triggered.

Some HyperPP patients with overlapping mutations with paramyotonia (PMC) showed myotonia and hypokalemic periodic paralysis successively in the same member, and both LET and SET were positive (Huang et al., 2019). Mexiletine can be induced by a small dose, but abnormal aggravation in hypokalemia should be vigilant.

### Andersen-Tawil syndrome

Andersen-Tawil syndrome (ATS) is an autosomal dominant disorder with incomplete penetrance. Most patients have KCNJ2 mutations that encode the inward rectifier potassium channel Kir2.1 (Binda et al., 2018; Plaster et al., 2001; Smith, Fish & Kannankeril, 2006), commonly referred to as ATS1, with common mutation loci *R218W, R218Q, R67W,* and *T192A*. The other proportion of ATS—with as yet unidentified mutated genes—is classified as ATS2. Most of the *KCNJ2* mutations in ATS1 result in the loss of function and dominant-negative inhibition of the Kir2.1 channel, culminating in a reduction in IK1 (Ai et al., 2002; Kuroda et al., 2017). IK1, which presents in human ventricular and atrial cardiomyocytes, may play an essential role in repolarizing action potentials and stabilizing resting potentials in humans. Recently, a mutation in *KCNJ5* encoding a G protein that activates the inward rectifier potassium channel Kir3.4 was identified in a patient diagnosed with preexisting ATS (Kokunai et al., 2014). However, the number of cases of *KCNJ5* mutations in *KCNJ2*-negative ATS disorders must yet be investigated, as must its relationship with other genes.

Kir2.1 and Kir3.4 are members of the inward rectifier potassium channel J subfamily that also includes *KCNJ4* (Bond et al., 1994). Kir2.1 is expressed in the heart, skeletal muscles, and brain, while Kir3.4 is expressed in skeletal muscles and heart tissues, although its physiological role in the former is unknown. Compared to other cation channels, the potassium outflow at the potential close to resting level by inward rectifier potassium channels is physiologically significant in the late repolarization phase for the stabilization of membrane potential, allowing for effective depolarization, for once a certain depolarization threshold is reached, the conductance drops rapidly without shunting potassium. In practically all literature reports, *KCNJ2* mutations cause loss of function, resulting in the dominant-negative inhibition of the Kir2.1 channel function due to the co-assembly of wild-type and mutant subunits 5 (Adams et al., 2016; Lu et al., 2006). *KCNJ5* encodes a G protein-sensitive inward rectifier potassium channel, with the *β*-subunit of the G protein activating *KCNJ3* and *KCNJ2* through direct interaction with its cytoplasmic N- and C-termini. Mutant subunit Kir3.4 has a significant negative effect on inwardly rectifying potassium currents *in vitro* when co-expressed with Kir2.1 in the same vector (Kokunai et al., 2014).

The mechanism of ATS is that *KCNJ2* (or *KCNJ5*) deficiency causes Kir2.1 current reduction, slow repolarization and prolonged action potential. The electrophysiological features were repetitive discharges on electromyography (EMG), polymorphic ventricular tachycardia or long QT on electrocardiogram (ECG), and positive LET with mild decrease (20–30%) (Jin et al., 2017; Kukla et al., 2014; Ribeiro et al., 2022; Simmons et al., 2019; Zhang et al., 2005). The clinical features were hyperkalemia, hypokalemia, normal serum potassium, and skeletal deformities (syndactyly, micrognathia).

### Thyrotoxic periodic paralysis

The combination of genetic susceptibility and hyperthyroidism alone is thought to be the trigger of TPP. Although TPP is clinically similar to familial HypoPP, mutations in the voltage-gated skeletal sodium channel Nav1.4 (*SCN4A*), the calcium channel Cav1.1 (*CACNA1S*), and the less frequent inward rectifier potassium channel Kir2.1 (*KCNJ2*) in TPP patients have not been uncovered (Hoi-Yee Li et al., 2020; Kung et al., 2004; Ng et al., 2004), whereas, single nucleotide polymorphism (SNP) has been identified in the *CACNA1S* region (encoded as Cav1.1). Nevertheless, the expression of calcium channels could alter the action of thyroid hormones. Several studies have demonstrated that mutations in *KCNJ18*, which encodes the potassium channel Kir2.6, are associated with TPP (Barski et al., 2009; Berwaerts et al., 1996; Gbefon et al., 2021; Nida & Kanj, 1998; Noso et al., 2019; Ovadia et al., 2006; Pannu & Sharma, 2019; Seshadri, Frank & Iqbal, 2002; Sonkar, Kumar & Singh, 2018; Villar Jimenez et al., 2013). *KCNJ18* gene mutations in patients with TPP are *K366R, R205H*, *Q407X*, *T354M*, *R339X*, *K360T*, *Q126X*, *E388K*, and *A200P*.

Kir2.6 binds to other Kir2.x subunits, such as Kir2.1, to form homotetrameric complexes capable of altering the inward transportation of potassium ions, thereby affecting the stability of the resting membrane potential. Reducing the inward flow of potassium ions predisposes the muscle membrane to abnormal depolarization and exposes it to muscle paralysis (Lior et al., 2011; Paninka et al., 2017). Kir2.6 contains a thyroid response element that can regulate gene transcription. Increased Na^+^/K^+^-ATPase activity could result in hypokalemia in TPP patients with hyperthyroidism, with the levels and activity of Na^+^/K^+^-ATPase having been shown to increase in TPP patients compared to a healthy control group and non-TPP patients with primary hyperthyroidism (Batra et al., 2023). This occurrence is due to the direct effect of hyperthyroidism and the impact of increased *β*2-adrenoceptor expression (Harrison & Clausen, 1998). In addition, insulin, epinephrine stimulation, androgens, or physical exercise could further escalate Na^+^/K^+^-ATPase activity, which explains why these factors render TPP patients prone to attacks (Hsieh et al., 2008; Yeh et al., 2014). An impaired potassium outflow instigated by mutations in inward rectifier potassium channels alongside increased Na^+^/K^+^-ATPase activity could induce abnormal depolarization and skeletal muscle non-excitability, ultimately triggering episodes of weakness.

### Other periodic paralysis-associated genes

PP forms are associated with mutations in the mitochondrial MTATP6/8 gene (Aure et al., 2013; Panosyan, Tawil & Herrmann , 2017), with patients presenting with episodic weakness. *In vitro* experiments have revealed the constant depolarization of the cell membrane, which perhaps is related to the impaired activity of ATP-dependent K^+^ channels. In one report, a patient with mutations in the Ryanodine receptor gene RYR1 developed intermittent weakness at 19 years old (Zhou et al., 2010), which initially affected his legs before spreading to the upper limbs and persisting for several days, with normal respiratory and sphincter function. The symptoms manifested were enhanced potassium-preserving diuretics. Genetic sequencing identified mutations in *Arg2241X*, *Asp708Asn*, and *Arg2939Lys*. *In vivo* RyR1 protein expression and Cav1.1 distribution in the muscle were abnormal, but RyR1 functioning and RyR1/Cav1.1 co-localization were normal; however, the precise mechanism of the episodes of weakness was unexplained.

In another report, a pediatric patient negative for all known periodic paralysis genes, but with hypokalemic PP and CNS (Sampedro Castañeda et al., 2018), was also found to have an *ATP1A2* mutation. A novel heterozygous missense mutation was identified in the *ATP1A2* gene encoding the Na^+^/K^+^-ATPase *α*2 subunit and expressed abundantly in skeletal muscles and brain astrocytes. Electrophysiological measurements established a low turnover of extracellular K^+^ And abnormal internal leakage currents were observed under reduced potassium conditions, prompting skeletal muscle depolarization. This finding supports leakage currents as a key pathological mechanism of hypokalemic PP and suggests *ATP1A2* as a practical new gene for hypokalemic PP.

### Nav1.4 mutations with overlapping phenotypes

A new heterozygous mutation, p. *Ala 204Glu (A204E)*, in Nav1.4 with clinical symptoms of HypoPP and HyperPP episodes of muscle paralysis has been identified (Kokunai et al., 2018). In functional analysis, *A204E* significantly decreased sodium current density, increased window currents, enhanced fast and slow inactivation of Nav1.4, and did not induce gating pore currents. Additionally, low extracellular K^+^ concentrations enhanced the negative impact of *A204E* on Nav1.4 activation. The Nav1.4 mutation, *p.R1451L*, located in VSD-IV, has also been reported in related literature (Luo et al., 2018). Heterozygous carriers of both lines present myotonia and HypoPP or HyperPP; in contrast, homozygous cases present HypoPP and myotonia, with functional analysis showing reduced current density and an enhanced closed-state inactivation of the mutant channels but no gating pore current phenomenon.

## Conclusions

In the study of the genetics of PP, analyzing gene mutations assists with the accurate diagnosis, prevention, treatment, and genetic counseling of weakness attacks. Also, exploring the pathogenesis of different gene mutation types of PP contributes to instructing clinical medication and aids in predicting disease attacks. The development of molecular biology techniques, the collection of clinical data on PPs, and further investigation on genetics have seen more and more unknown gene mutations identified, and the understanding of their pathogenesis has become more comprehensive and accurate, providing a crucial basis for the better diagnosis and treatment of PP and offers guidance for genetic counseling and the prognosis of PP.

## Additional Information and Declarations

### Data Availability

The following information was supplied regarding data availability: This article is a literature review.
